# Risk Factors of Postoperative Hospital-Acquired Pneumonia in Patients Undergoing Cardiac Surgery

**DOI:** 10.3390/medicina59111993

**Published:** 2023-11-13

**Authors:** Piotr Duchnowski, Witold Śmigielski

**Affiliations:** Ambulatory Care Unit, Cardinal Wyszynski National Institute of Cardiology, 04-628 Warsaw, Poland

**Keywords:** high-sensitivity Troponin T (hs-TnT), right ventricular systolic pressure (RVSP), valve surgery, hospital-acquired pneumonia

## Abstract

*Background and Objectives*. Hospital-acquired pneumonia is one of the complications that may occur in the postoperative period in patients undergoing heart valve surgery, which may result in prolonged hospitalization, development of respiratory failure requiring mechanical ventilation or even death. This study investigated the preoperative risk factors of postoperative pneumonia after heart valve surgery. *Materials and Methods*: This was a prospective study in a group of consecutive patients with hemodynamically significant valvular heart disease undergoing valve surgery. The primary endpoint at the in-hospital follow-up was hospital-acquired pneumonia after heart valve surgery. Logistic regression analysis was used to assess which variables were predictive of the primary endpoint, and odds ratios (ORdis) were calculated with a 95% confidence interval (CI). Multivariate analysis was based on the results of single-factor logistic regression, i.e., in further steps all statistically significant variables were taken into consideration. *Results*: The present study included 505 patients. Postoperative pneumonia occurred in 23 patients. The mean time to diagnosis of pneumonia was approximately 3 days after heart valve surgery (±2 days). In multivariate analysis, preoperative level of high-sensitivity Troponin T (hs-TnT) (OR 2.086; 95% CI 1.211–3.593; *p* = 0.008) and right ventricular systolic pressure (RVSP) (OR 1.043; 95% CI 1.018–1.067; p 0.004) remained independent predictors of the postoperative pneumonia. Of the patients with postoperative pneumonia, 3 patients died due to the development of multiple organ dysfunction syndrome (MODS). *Conclusions*: Preoperative determination of serum hs-TnT concentration and echocardiographic measurement of the RVSP parameter may be useful in predicting postoperative pneumonia in patients undergoing heart valve surgery.

## 1. Introduction

Hospital-acquired pneumonia is one of the complications that may occur in the postoperative period in patients undergoing heart valve surgery, which may result in prolonged hospitalization, development of respiratory failure requiring mechanical ventilation, multiple organ dysfunction syndrome or even death [1,2,3,4,5]. By definition, hospital-acquired pneumonia is a pneumonia that occurs forty-eight hours after admission to hospital in a patient who was not intubated at the time of admission. Ventilation-associated pneumonia (VAP) is pneumonia that occurs >48 h after the initiation of invasive mechanical ventilation. The etiological factors of hospital-acquired pneumonia during the first 4 days of hospitalization are the same bacteria and viruses that cause community-acquired pneumonia, including gram-negative bacilli (*E. coli*, *K. pneumoniae*, *Enterobacter*, *Proteus* and *Serratia*), but with preserved sensitivity to antibiotics. In turn, from the 5th day of hospitalization, multidrug-resistant (MDR) strains predominate, most often aerobic gram-negative bacilli: *P. aeruginosa*, *E. coli*, *K. pneumoniae*, *Acinetobacter* spp., and *L. pneumophila*, and among the gram-positive bacteria mainly *S. aureus*, of which hospital strains may be resistant to methicillin. The bacterial flora and its resistance to antibiotics differs between hospitals, so each hospital should develop its own profile of microorganisms causing nosocomial infections along with their drug sensitivity (separately for the intensive care unit). Sources of microorganisms include healthcare devices, the environment (air, water, equipment, and clothing) and the transmission of microorganisms between the patient and staff or other sick people. Risk factors for infection with MDR bacteria causing VAP include: intravenous antibiotic therapy in the last 90 days; septic shock; ARDS; duration of current hospitalization ≥5 days; and renal replacement therapy for acute indications before the appearance of VAP symptoms. Risk factors for infection with MDR strains of *P. aeruginosa* and other intestinal bacteria include: a stay in an intensive care unit where >10% of strains of gram-negative bacteria causing VAP may be resistant to an antibiotic used in monotherapy or the sensitivity of the local flora to antibiotics is unknown; and colonization or previous isolation from a patient with *P. aeruginosa* or other gram-negative intestinal bacteria. In turn, risk factors for MRSA infection include staying in a ward where >10–20% of *S. aureus* strains causing VAP are resistant to methicillin or the frequency of isolation of MRSA strains is unknown, and colonization or previous isolation of MRSA from the patient [6,7,8,9,10,11].

Troponin T (TnT) is a protein that is part of the contractile apparatus of striated muscles. TnT functions in all types of striated muscles are the same, but cardiac TnT (cTnT) differs from TnT found in skeletal muscle. Therefore, cTnT detected in plasma is a highly specific marker of myocardial damage (necrosis). Highly sensitive troponin tests have been available for several years and detect even minimum levels of Troponin T in blood serum with great credibility. Currently, cTnT is a recognized biomarker of myocardial damage, widely used in the diagnosis and prognosis of patients with cardiovascular diseases such as myocardial infarction, chronic coronary syndrome, pulmonary embolism, and postoperative complications such as postoperative cardiogenic shock [12]. 

On the other hand, pulmonary hypertension is defined as an increase in mean pulmonary artery pressure (mPAP) ≥ 20 mm Hg at rest, marked by right heart catheterization (RHC). One of the important and relatively common causes of pulmonary hypertension is severe valvular disease in the left side of the heart. The recognized imaging method used to estimate the right ventricular systolic pressure (RVSP) is continuous-wave Doppler echocardiography [13,14,15,16,17]. To date, numerous publications have shown that pulmonary hypertension is a predictor of numerous cardiovascular complications and postoperative mortality [18,19,20,21,22,23].

Knowing the predictors of postoperative hospital-acquired pneumonia allows you to improve patient outcomes with valvular heart disease by choosing the optimal time interval for surgical treatment, special supervision of high-risk patients in the perioperative period, and, in the event of the first symptoms of pneumonia, implementing early diagnostics and treatment. 

Therefore, in the present study, the authors attempted to evaluate selected preoperative parameters in terms of their ability to predict the occurrence of postoperative hospital-acquired pneumonia in patients undergoing cardiac surgery due to valvular heart disease.

## 2. Methods

A prospective study was carried out on a group of consecutive patients with hemodynamically significant valvular heart disease (aortic stenosis, aortic regurgitation, mitral stenosis, and mitral regurgitation) treated with a classic cardiac surgical method at the Cardinal Wyszynski National Institute of Cardiology in Warsaw, Poland. Exclusion criteria: no signed consent to participate in the study, age less than 18 years, presence of porcelain aorta, significant stenoses in the cephalic arteries, active diagnosed cancer, active autoimmune diseases. Before the cardiac surgery, blood was taken from each patient to determine selected laboratory parameters. A Cobas 6000 electronic counter (Roche, Germany) was used for complete blood counts. In turn, the troponin T hs-STAT test (Roche) was used to assess the level of cardiac Troponin T in blood serum. Systolic pressure in the right ventricle was estimated using the continuous-wave Doppler method of transthoracic echocardiography, based on the measurement of the maximum wave velocity of tricuspid regurgitation, according to the simplified Bernoulli equation, adding to the obtained result the pressure in the right atrium. The right atrial (RA) pressure was determined by means of echocardiography based on the diameter and respiratory tract diameter of the inferior vena cava (IVC): IVC diameter < 2.1 cm, which collapses > 50% with rapid breathing through the nose, indicates normal pressure in RA of approximately 3 mm Hg, while the diameter IVC > 2.1 cm, which decreases <50% when breathing rapidly through the nose or <20% when breathing in calmly, indicates a high pressure in the RA within 15 mmHg. In situations in which the diameter IVC and the extent of collapsing its diameter did not match the above-mentioned scheme, an intermediate value of 8 mm Hg was adopted. The heart valve surgery was performed through a midline sternotomy incision under general anesthesia and normothermic conditions, with the use of extracorporeal circulation. Each patient received a prophylactic dose of the antibiotic cefazolin at a dose of 2 g 30 min before the first surgical incision.

The primary endpoint was the diagnosis of hospital-acquired postoperative pneumonia. The diagnosis of hospital-acquired pneumonia was made in the case of the appearance of new or progression of existing lung infiltrates on imaging tests such as chest X-rays and computed tomography and the presence of ≥2 of 3 clinical criteria: body temperature ≥38 °C; leukocytosis or leukopenia; and/or purulent secretion in the bronchi (increase in the amount of secretion or change in its nature to purulent). In all patients with suspected hospital-acquired pneumonia, blood was collected for culture and/or samples of secretions from the lower respiratory tract were collected (aspiration from the tracheal lumen with semi-quantitative culture or bronchoscopic methods with quantitative culture). Material for microbiological tests was collected before the initiation of empirical antibiotic therapy. The total follow-up period of patients included in the study was until discharge from the hospital or the date of death. The study was conducted at Cardinal Wyszynski National Institute of Cardiology, Warsaw, Poland, after prior approval of the study protocol by the Institutional Ethics Committee, study number 2.32/VI/18.

### Statistical Analysis

Data are presented as the mean ± standard deviation (SD) and the frequency (%). Logistic regression analysis was used to asses which variables were predictive of primary endpoint, and odds ratios (ORdis) were calculated with a 95% confidence interval (CI). Multivariate analysis was based on results of single-factor logistic regression, i.e., in further steps all statistically significant variables were taken into consideration. To improve the interpretation of odds ratio, all quantitative variables were recoded to binary variables based on cut-off points established by using the ROC curves. Statistical data analyses were performed using STATISTICA software (StatSoft Polska Sp. z o.o.; Kraków, Poland). Statistical significance was defined as *p* < 0.05 for all the analyses.

## 3. Results

The study included 505 patients undergoing valve(s) repair or replacement at the National Institute of Cardiology in Warsaw, Poland, a top-tier center. The mean age in the studied population was 63 (standard deviation [SD] ± 12). One hundred and ten patients (21% of the study group) had a history of active nicotine addiction, while thirty-five patients (7% of all patients included in the study) were diagnosed with chronic obstructive pulmonary disease. The mean preoperative right ventricular systolic pressure level was 41 ± 17 mm Hg and the preoperative hs-TnT level was 36 ± 25 ng/L. Table 1 shows the characteristics of the patients studied. The postoperative hospital-acquired pneumonia occurred in 23 patients. The mean time to diagnosis of pneumonia was approximately 3 days after heart valve surgery (± 2 days). The mean extubation time from the end of surgery in twenty-two patients diagnosed with postoperative pneumonia was 6 h on average (SD ± 4), while one patient who developed postoperative pneumonia was mechanically ventilated due to circulatory and respiratory failure for 4 days. Only in 4 cases was the pathogen responsible for pneumonia identified on the basis of a microbiological blood culture and/or a bronchoalveolar lavage (BAL) test (*P. aeruginosa* in one patient, vancomycin-resistant *Enterococcus* in one patient, *Methicillin-susceptible Staphylococcus aureus* in one patient, and *Enterobacter cloacae* in one patient). In each case, after the diagnosis of pneumonia and the collection of material for microbiological tests, empirical antibiotic therapy was started. As a result of showing no clinical improvement, the antibiotic therapy was changed in 5 patients, including 3 patients after obtaining a positive microbiological test result. For every patient diagnosed with pneumonia, chest radiography showed compaction in one or both pulmonary fields. The average value of the CRP parameter at the time of diagnosis of pneumonia was 12 ± 7 mg/dl and the white blood cell count was 15 ± 6 × 1000/uL. Due to the severity of the symptoms of respiratory failure, 8 patients with postoperative pneumonia required re-intubation and initiation of mechanical ventilation, of which 3 patients died. Table 2 presents statistically significant predictors of postoperative pneumonia identified in univariate analysis. In multivariate analysis, the preoperative levels of high-sensitivity Troponin T (hs-TnT) (OR 2.086; 95% CI 1.211–3.593; *p* = 0.008) and right ventricular systolic pressure (RVSP) (OR 1.043; 95% CI 1.018–1.067; *p* = 0.004) remained independent predictors of the primary endpoint. None of the comorbidities, including the presence of chronic obstructive pulmonary disease and the history of active nicotine addiction, reached statistical significance for the primary endpoint in the univariate logistic regression analysis. The real 30-day mortality was 3.7 vs. 3.0 expected mortality calculated using EuroSCORE II (www.euroscore.org).

## 4. Discussion

Hospital-acquired pneumonia is a serious complication that may occur in the early postoperative period in patients undergoing heart valve/valves surgery, carrying a significant risk of developing respiratory failure, multiple organ failure and death. Our study showed that this complication occurred in 4.5% of patients after heart valve surgery. Pneumonia is most often caused by microorganisms, including bacteria. Thanks to the body’s natural defense mechanisms, the lower respiratory tract remains sterile under physiological conditions. It happens that microbes get into the lower respiratory tract—usually by aspiration. Bacteria and other microorganisms are then killed by the cells of the immune system. However, it sometimes happens that, under favorable conditions, microorganisms can survive in the lower respiratory tract, which stimulates the body to turn on defense mechanisms. Then, within the lung parenchyma, an inflammatory process develops, which results in an increase in the permeability of the walls of the blood vessels surrounding the alveoli. The effect of this is, among others, accumulation of purulent contents in the alveoli and small bronchioles, consisting of microorganisms, neutrophils, and remnants of dead cells. The result of these processes is the loss of physiological functions, mainly gas exchange of the alveoli involved in the inflammatory process [24,25,26,27,28]. In the case of extensive changes in the lungs, the patient feels severe shortness of breath, and features of respiratory failure develop, which can lead to tissue hypoxia and may have further adverse consequences. Therefore, the development of hospital-acquired pneumonia in the early postoperative period carries a significant risk of prolonged hospitalization, development of respiratory failure, the need for re-intubation and the use of mechanical ventilation, and death. Considering the above, knowledge about the predictors of hospital-acquired pneumonia is extremely important, because it enables preoperative identification of patients at risk of postoperative pneumonia, indicating the optimal time of surgery and the need for special supervision of the patient in the perioperative period, and enables the implementation of early diagnosis and treatment in the event of the first symptoms of inflammation development. In the presented study conducted on a group of 505 patients undergoing heart valve surgery, the preoperative levels of hs-TnT and RVSP assessed before cardiac surgery using echocardiography were independent predictors of postoperative pneumonia. It is worth noting that the statistical analyses performed showed that none of the comorbid chronic diseases, including chronic obstructive pulmonary disease or a history of active nicotine addiction, achieved statistical significance in terms of the predictive ability of postoperative pneumonia.

Troponin T (TnT) is a protein that is part of the contractile apparatus of striated muscles. The function of TnT in all types of striated muscle is the same, but cardiac TnT differs from TnT found in skeletal muscle [29]. Therefore, cTnT present in the blood is a highly specific marker of myocardial damage. In patients with severe valvular heart disease, pressure and/or volume overload of the heart muscle develops. A long-term increase in the tension of the walls of the left ventricle leads to their overload and may lead to progressive damage to cardiomyocytes, the slow development of necrosis and myocardial fibrosis. This is due, among other things, to a decrease in myocardial perfusion, mainly in the subendocardial layer [30,31]. The result of pathological processes taking place in the overloaded left ventricular muscle is a persistently elevated level of troponin T in plasma and the development of heart failure, which may be manifested by persistent blood stasis in the pulmonary circulation. 

On the other hand, the presence of severe valvular heart disease in the left side of the heart is one of the causes of increasing pressure in the pulmonary artery [13,24,25]. Right heart catheterization (RHC) has become the established method of assessing pulmonary artery pressure. However, it is a costly, invasive, difficult to access and time-consuming method. Therefore, the use of other non-invasive diagnostic methods evaluating systolic pressure in the right ventricle, such as transthoracic echocardiography despite its limitations, is currently very helpful in assessing the likelihood of pulmonary hypertension [13,14,15,16,27]. The long-term presence of elevated values of pressure in the pulmonary circulation results in the development of various unfavorable pathophysiological mechanisms, which include vasoconstriction, pulmonary vascular remodeling with proliferation and narrowing of the light, and inflammation as well as thrombosis. The effect of prolonged increased pressure within the pulmonary vessels may be the development of endothelial dysfunction, which in turn favors the reduction in the production of important antiproliferative substances, such as prostacyclin and nitric oxide. Dysfunction of the pulmonary vascular endothelium is favored by overexpression of vasoconstrictive and proliferative compounds, such as thromboxane and endothelin 1. The effect of these pathomechanisms is a further increase in pulmonary vascular resistance and abnormal remodeling of the vascular wall, including the proliferation of endothelial cells, smooth muscles, and fibroblasts. There is also an increase in the extracellular matrix in terms of collagen, elastin, fibronectin and tenascin, and the influx of inflammatory cells [26,27,28]. It seems, therefore, that the pathophysiological processes occurring in the lung tissue of patients with persistently elevated pulmonary artery pressure in the course of long-term valvular disease, as well as persistent stasis in the course of heart failure, may favor the development of pneumonia, especially in the critical period of stay in the intensive care unit in the immediate period after heart valve surgery. 

The results of the presented study may therefore indicate that the respiratory system in a patient with an overloaded circulatory system in the course of valvular heart disease, as expressed by the above values of Troponin T and pulmonary pressure, is particularly sensitive to unfavorable conditions in the perioperative period, including the need for intubation and the use of mechanical ventilation during surgery and the stay in the postoperative ward, or the administration of intravenous drugs, including blood transfusion, which favor contamination of the respiratory system with microorganisms. Therefore, taking into account the results of the above study, as well as numerous previous studies, it should be emphasized that periodic control of markers of heart muscle damage such as troponin T and NT-proBNP and, moreover, pulmonary pressure, e.g., using the echocardiographic RVSP parameter, may be very useful in the observation of patients with heart valve disease from the moment of diagnosis. It seems that the choice of the optimal date of cardiac surgery in patients with severe valvular heart disease may be decisive in order to protect the patient against an increased risk of serious postoperative complications [25,32,33]. Perhaps earlier qualification for surgical treatment of valvular heart disease in a patient with less advanced myocardial damage in the course of valvular heart disease, expressed, among others, by lower values of parameters such as Troponin T or NT-proBNP, and lower values of pulmonary pressure, may stop the unfavorable pathophysiological mechanisms occurring both in the left ventricular muscle and in the lung tissue, and thus may reduce the risk of developing unfavorable postoperative complications. In addition, knowledge of the predictors of postoperative pneumonia may also facilitate the planning of cardiac surgery in a patient with overloaded heart muscle and high RVSP values, among others, by selecting an experienced team of specialists, rigorous adherence to asepsis in the perioperative period, and extremely careful monitoring of the early postoperative course, which in turn may protect the patient from pneumonia and improve treatment outcomes for patients with valvular heart disease.

## 5. Summary

The results of this study indicate that determination of hs-TnT and RVSP levels during the period may be useful in predicting postoperative pneumonia. In addition, they may suggest that regular control of the above-mentioned parameters may be useful when managing patients with valvular heart disease in order to assess myocardial overload and to identify the optimal moment to qualify for interventional treatment of valvular heart disease. This is a single-center study with a limited number of patients. Further research is needed to elucidate the pathomechanisms linking the increased risk of postoperative pneumonia in patients with higher RVSP and hs-TnT levels. The results of our research may be helpful in developing a perioperative strategy in patients undergoing cardiac surgery. It is also worth noting that the study did not assess the Charlson comorbidity index, and therefore it was not used in the logistic regression analyses. Nevertheless, none of the comorbidities listed in Table 1 presenting patient characteristics achieved statistical significance.

## Figures and Tables

**Table 1:** Baseline characteristics of the study population.

Preoperative Characteristics of Patients (*n* = 505)	Values
Age, years *	63 ± 12
Male: men, *n* (%)	285 (56)
Body mass index, kg/m^2^ *	28 ± 11
LV ejection fraction, (%) *	55 ± 11
Right ventricular systolic pressure (RVSP), mm Hg *	41 ± 17
EuroSCORE II, %*	3.0 ± 1.9
Atrial fibrillation, *n* (%)	271 (42)
Chronic kidney disease (GFR < 60 mL/min/1.73 m^2^), *n* (%)	167 (33)
Coronary artery disease, *n* (%)	76 (16)
Previous myocardial infarction, *n* (%)	50 (9)
Stroke history, *n* (%)	40 (7)
Hypertension, *n* (%)	328 (64)
Diabetes mellitus, *n* (%)	96 (19)
Current smoker, *n* (%)	110 (21)
Chronic obstructive pulmonary disease, *n* (%)	35 (7)
Hematocrit, (%) *	41 ± 5
Hemoglobin, g/dL *	13.6 ± 1.4
RDW, (%) *	14.1 ± 0.8
GFR, mmol/L *	67 ± 16
Hs-TnT, ng/L *	36 ± 25
NT-proBNP, pg/mL	1923 ± 1433
CRP, mg/dL	0.4 ± 0.2
Platelets, 1000/uL*	192 ± 55
**Main procedures:**	
AVR, *n* (%)	260 (51)
AVP, *n* (%)	12 (2)
AVR + MVR, *n* (%)	52 (10)
AVR + MVP, *n* (%)	5 (1)
AVP + MVP, *n* (%)	9 (2)
MVP, *n* (%)	78 (15)
MVR, *n* (%)	89 (17)
**Concomitant procedures:**	
CABG, *n* (%)	77 (15)

The values are represented by the mean * and a measure of the variation in the internal standard deviation. Abbreviations: AVR = aortic valve replacement, AVP = aortic valve plasty, CABG = coronary artery bypass graft, CRP = C-reactive protein, GFR = glomerular filtration rate, Hs-TnT = high sensitivity troponin t, LV = left ventricle, MVR = mitral valve replacement, MVP = mitral valve plasty, NT-proBNP = n-terminal of the prohormone brain natriuretic peptide, RVSP = right ventricular systolic pressure.

**Table 2:** Analysis of predictive factors for the occurrence of postoperative hospital-acquired pneumonia.

	Univariate Analysis			Multivariate Analysis		
Variable	Odds Ratio	95% Cl	*p*-Value	Odds Ratio	95% Cl	*p*-Value
Hemoglobin, g/dL	0.711	0.551–0.919	0.009			
Hs-TnT, ng/L	1.778	1.248–2.535	0.001	2.086	1.211–3.593	0.008
NT-proBNP, pg/mL	1.475	1.067–2.041	0.01			
RVSP, mm Hg	1.029	1.007–1.052	0.008	1.303	1.007–1.061	0.01

Abbreviations: Hs-TnT = high sensitivity troponin t, NT-proBNP = n-terminal of the prohormone brain natriuretic peptide, RVSP = right ventricular systolic pressure.

## Data Availability

Research data available from the author of the publication.
